# An automated real-time microfluidic platform to probe single NK cell heterogeneity and cytotoxicity on-chip

**DOI:** 10.1038/s41598-021-96609-9

**Published:** 2021-08-24

**Authors:** Nikita Subedi, Laura C. Van Eyndhoven, Ayla M. Hokke, Lars Houben, Mark C. Van Turnhout, Carlijn V. C. Bouten, Klaus Eyer, Jurjen Tel

**Affiliations:** 1 Laboratory of Immunoengineering, Department Biomedical Engineering, Eindhoven University of Technology, Eindhoven, The Netherlands; 2 Institute for Complex Molecular Systems (ICMS), Eindhoven University of Technology, Eindhoven, The Netherlands; 3 Soft Tissue Engineering and Mechanobiology (STEM), Department Biomedical Engineering, Eindhoven University of Technology, Eindhoven, The Netherlands; 4 Laboratory for Functional Immune Repertoire Analysis, Institute of Pharmaceutical Sciences, D-CHAB, ETH Zürich, Zurich, Switzerland

**Keywords:** Immunology, Cell death and immune response

## Abstract

Cytotoxicity is a vital effector mechanism used by immune cells to combat pathogens and cancer cells. While conventional cytotoxicity assays rely on averaged end-point measures, crucial insights on the dynamics and heterogeneity of effector and target cell interactions cannot be extracted, emphasizing the need for dynamic single-cell analysis. Here, we present a fully automated droplet-based microfluidic platform that allowed the real-time monitoring of effector-target cell interactions and killing, allowing the screening of over 60,000 droplets identifying 2000 individual cellular interactions monitored over 10 h. During the course of incubation, we observed that the dynamics of cytotoxicity within the Natural Killer (NK) cell population varies significantly over the time. Around 20% of the total NK cells in droplets showed positive cytotoxicity against paired K562 cells, most of which was exhibited within first 4 h of cellular interaction. Using our single cell analysis platform, we demonstrated that the population of NK cells is composed of individual cells with different strength in their effector functions, a behavior masked in conventional studies. Moreover, the versatility of our platform will allow the dynamic and resolved study of interactions between immune cell types and the finding and characterization of functional sub-populations, opening novel ways towards both fundamental and translational research.

## Introduction

The human immune system operates in an intricate environment that involves a complex and coordinated system to identify and eliminate threats. Since both pathogens and tumour cells can be highly variable, the immune system evolved into a highly heterogeneous, but tightly regulated system^1^. Traditionally, the diversity in immune cell populations is characterised predominantly by differences in morphology and expression of static markers. However, cellular effector functions, such as migration of immune cells to the site of infection, release of cytokines, or cytolytic activity, also exhibit enormous variation between and among well-defined cellular subsets, such as the cytolytic heterogeneity observed in NK cells^2,3^. Although several studies have highlighted how heterogeneity is a hallmark of immune system and plays an important role in shaping the overall immune response, the functional aspects of cellular heterogeneity remain largely unexplored^4^. So far, the majority of both in vivo animal models and in vitro experiments mainly only yielded an averaged outcome as a consequence of the interactions between multiple cells, rather than providing functional readouts of single-activated cells. Additionally, the current systems that can provide functional readout of individual cells either lack throughput or fail to isolate individual cell pairs to exclude possible effects of paracrine signalling. Since a well-functioning immune response is the result of combined efforts of multiple, and diverse individual cells, understanding heterogeneity within immune cell populations is crucial for a better understanding of the overall immune response and the effector functions in a cell population.

Cytotoxicity is an important effector mechanism used by Cytotoxic T cells (CTLs) and Natural Killer (NK) cells to combat both pathogens and tumour cells. Cytotoxicity assays are essential to understand the functional activation and cytolytic impact of individual effector cells towards various target cells, including heterogenous tumours. For decades, the gold standard to assess immune cell-mediated cytotoxicity used to be the chromium release assay, where the release of radioactive ^51^Cr by dying target cells is indicative for specific lysis^5^. While quantitative and precise, this method requires the handling and disposal of radioactive compounds. Recently, several other alternative approaches have been developed, e.g. fluorescent probes to detect cell viability^6^, metabolic MTT assay^7^, enzyme-based LDH assays^8^, and luciferase transduced cell-based bioluminescence assays^9^. Even though these assays have provided insight into fundamental cellular behaviour, these studies are performed in bulk and generate averaged responses^10^. Bulk-based studies fail to address the functional heterogeneity underlying a given cell population by masking the phenotype, gene expression, and the mechanism of cellular communication in between individual immune cells^11,12^. To overcome the limitations of bulk methodologies, the study of cytotoxicity at a single-cell level can be performed using flow cytometry-based assays and single cell microscopy assays^13,14^. However, flow cytometric assays are snapshot-based and therefore not suited to monitor temporal dynamics of cellular interactions leading to cytotoxicity^10^. Besides, single cell microscopy systems average out the overall population dynamics by allowing paracrine signalling to steer individual cell behaviors^1^.

Advances in technology and miniaturization yielded microsystem-based technologies that aim to overcome the limitations of conventional and flow cytometry-based assays^15,16^. These technologies enhanced the sensitivity of measurements, allowing the investigation of lower cell numbers using reduced reagent volumes. However, a major challenge of adopting microsystem technologies for interaction-based assays is the efficient pairing of effector and target cells^17^. Despite the numerous attempts to overcome this challenge, a novel approach that meets the requirements is a necessity.

Here we present a droplet-based microfluidic platform that, in conjunction with real-time fluorescence microscopy and an automated image analysis script, enables high-throughput monitoring of over 60,000 droplets therefore allowing to analyse around 20,000 droplets with cells. Droplet-based microfluidics is highly tuneable and provides the opportunity to pair cells in a compartmentalized and noise-free microenvironment^18–21^. The cellular encapsulation and pairing can be precisely controlled to achieve different effector:target ratios^22^. By studying the interaction between NK cells and different target tumour cells we present the possibility to study the underlying dynamics of cytotoxicity. Our data reveals heterogenous NK cell behaviour with a consistent percentage of the population displaying cytolytic abilities.

## Materials and methods

### Cell isolation and culture

K562 cells were cultured in 1:1 (v/v) mixture of RPMI 1640 (Gibco, Catalog no. 22400089) and IMDM (Gibco, Catalog no.12440053) supplemented with 10% fetal bovine serum (FBS; Gibco) and 1% penicillin/streptomycin (PS; Gibco). Jurkat T cells were cultured in RPMI, supplemented with 10% FBS and 1% PS. Both cell lines were regularly tested for mycoplasma contamination. Primary NK cells were obtained from buffy coats of healthy donors (Sanquin) after written informed consent according to the Declaration of Helsinki and all experimental protocols concur to institutional guidelines. In short, peripheral blood mononuclear cells (PBMCs) were isolated from donor blood via density gradient centrifugation using Lymphoprep Density Gradient Medium (Stem cell). The NK cells were subsequently isolated using magnet-activated cell sorting (MACS) by negative selection using the NK cell isolation kit (Miltenyi Biotech, Catalog no. 130-092-657) following the manufacturer’s instructions. Cells were counted and purity was routinely assessed using flow cytometry by cell surface marker staining for 10 min at 4 °C, using PE-CY7-labeled anti-CD56 (Biolegend, Catalog no. 362509), PE-labeled anti-CD16 (Biolegend, Catalog no. 302007), and PerCP-labeled anti CD3 (Biolegend, Catalog no. 300328) antibodies in 50 μL FACS buffer (2% FBS in PBS). The NK cells were identified as CD16^+^CD56^+^CD3^−^, and purity was on average 91%. Subsequently, isolated NK cells were encapsulated into droplets with K562 pair in presence of 1400 ng/mL IL2, as stimulant (Peprotech, Catalog no. 200-02) to monitor the cytotoxicity function of NK cells.

### Microfluidic chip for droplet production

**Figure 1:**
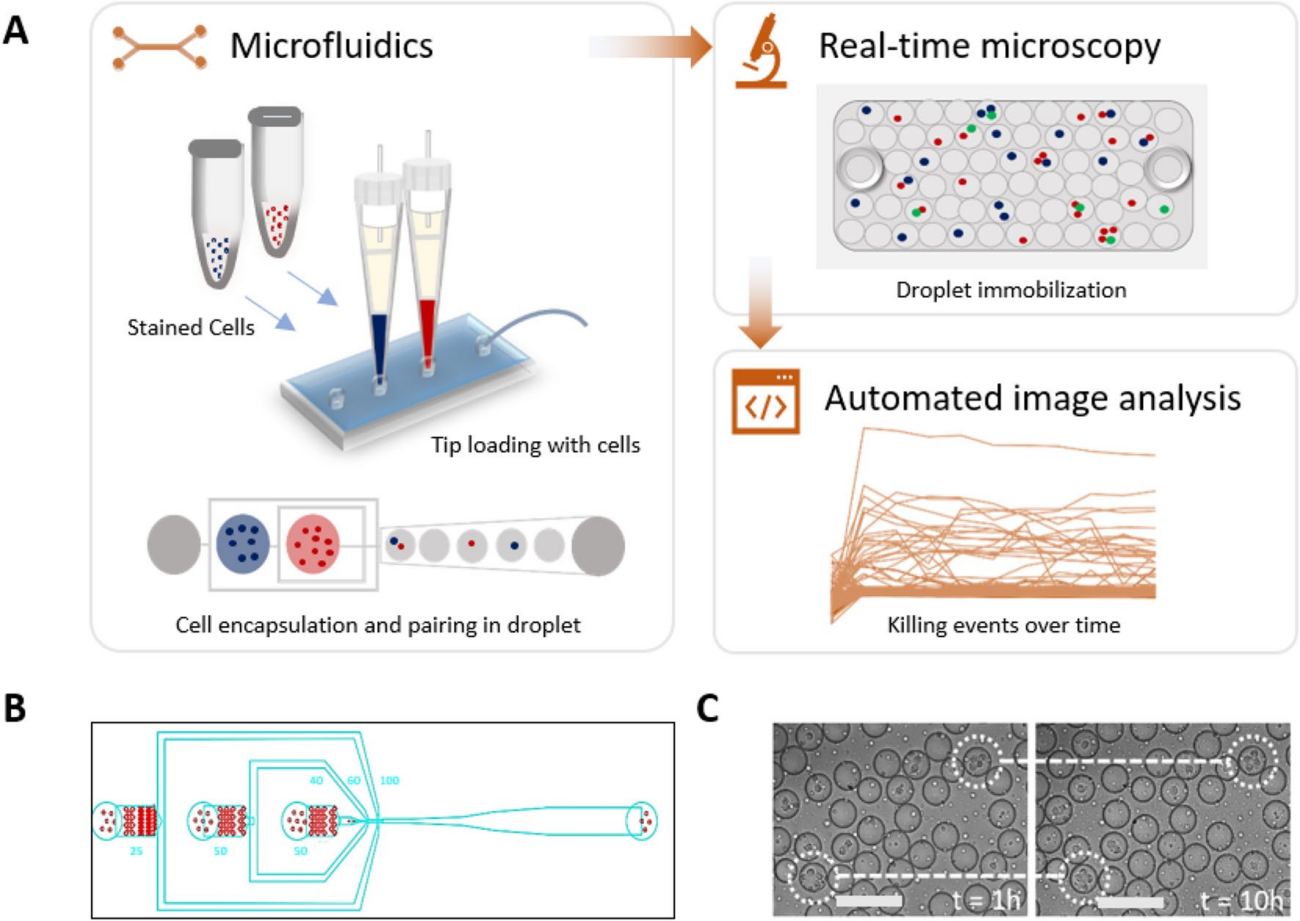
Experimental setup of high-throughput droplet-based cytotoxicity platform. (**A**) Experimental schematics showing cytotoxicity platform that combines (i) droplet generation and cell pairing using microfluidics, (ii) droplet immobilization for real-time microscopy, (iii) automated image analysis using custom-made MATLAB script to allow unbiased and high throughput detection of cytotoxic events. Stained NK cells and K562 cells were loaded into the chip using 200 µL pipette tips and encapsulated into droplets using a 3-inlet microfluidic chip. The viability dyes were included within the cell medium. The immobilized droplets were incubated in a stage top incubator set at 5% CO_2_ and 37 °C. Image acquisition was performed at every hour interval for 10 h. (**B**) The three-inlet microfluidic device with flow-focusing junction to generate droplets. (**C**) A qualitative test of the observation chamber was performed by monitoring droplets movement in the chamber under the microscope for 10 h.

The microfluidic device was molded using an SU-8 photo resist structure on a silicon wafer and a commercially available polydimethylsiloxane silicone elastomer (Sylgard 184, Dow Corning), mixed with curing agent at the ratio 10:1 (w/w) and allowed to cure for 3 h at 60 °C. The surface of the Sylgard 184 was activated by exposure to plasma and sealed with a plasma-treated glass cover slide to yield closed micro channels. Channels were subsequently treated with a 5% (v/v) silane (1H,1H,2H,2H-Perfluorooctyltriethoxysilane; Fluorochem, Catalog no. S13150) solution in fluorinated oil (Novec HFE7500, 3 M, Catalog no. 51243) and thermally bonded for 12 h at 60 °C. The dimensions of the microfluidic channels are 40 µm × 30 µm at the first inlet, 60 µm × 30 µm at the second inlet and the production nozzle, and 100 µm × 30 µm at the collection channel (Fig. 1B).

### Assembly of Droplet observation chamber

Glass microscopy slides (76 × 26 × 1 mm; Corning) were used as top and bottom covers (76 × 26 × 1 mm). Two access holes of 1.5 mm diameter were drilled in the top glass. Both slides were thoroughly cleaned using soap, water, and ethanol, and were exposed to air plasma (60 W) for 5 min. A cutout sheet of 60 μm thick double-sided tape (ORAFOL) was carefully placed above the bottom glass slide. Afterwards, the glass slides were stacked on top of each other and the assembly was pressed using Atlas Manual 15 T Hydraulic Press (Specac) for 5 min at 155 °C at 400 kg per m^2^ pressure load (Supplementary Fig. 1). Next, two nano ports (Idex) were attached to the holes using UV curable glue (Loctite 3221 Henkel) which was cured under UV light for 5 min. Subsequently, the surface of the 2D chamber was treated with 5% (v/v) silane solution. Lastly, the chamber was dried, filled with fluorinated oil, and sealed until used. The chamber was reused multiple times and cleaned after each experiment by flushing fluorinated oil to remove droplets and stored filled until the next use.

### Cell staining

5 µM Calcein Red AM (AAT Bioquest, Catalog no. 21900) and 10 µM Cell Tracker Blue dyes (Invitrogen, C2110) were used to stain primary NK cells and target cells (K562 or Jurkat), respectively. For staining, around 2 million cells were washed with RPMI free of supplements and Phenol red (Gibco, Catalog no. 11835030), resuspended in the freshly prepared dye solution in-RPMI (1 mL) and incubated at 37 °C for 30 min. Thereafter, cells were subsequently washed twice and resuspended in RPMI medium supplemented with 25 mM HEPES (Gibco, Catalog no. 15630056), 2% human serum (Sanquin), without Phenol red) containing 2.5 µM Sytox green (Invitrogen, Catalog no. S7020) and 7 µM CellEvent Caspase-3/7 Green Detection Reagent (Invitrogen, Catalog no. C10423), respectively. The stained cells were then proceeded for cell encapsulation.

### Cell loading in microfluidic chip

2.5 mL glass syringe and tubing polytetrafluoroethylene tubing were filled with biocompatible mineral oil (Sigma Aldrich, Catalog no. M8410-1L). The end of the tubing was attached to a PDMS plug of 5 µm diameter, which was then squeezed tightly into the large end of a pipette tip (200 µL). Subsequently, the pipette tip was filled with mineral oil from the syringe. To load cell suspension, the pipette tip was lowered into a cell solution and the syringe pump was used to aspirate the solution into the tip. Finally, the pipette tip was connected tightly to the inlet of a microfluidic chip and droplet production was started. The syringes were driven by computer-controlled syringe pumps (Nemesys, Cetoni GmbH). The droplets of ~ 50 µm diameter were generated using flow speed of 30 µL/min for oil and 5 µL/min for each sample inlet. The droplets were produced for around 5 min, thus generating 700,000 droplets in total. For the stability of droplets, 2.5% (v/v) Pico-Surf surfactant (Sphere Fluidics, Catalog no. C024) was used in fluorinated oil.

### Single NK cell cytotoxicity assay

Primary NK cells and target cells were loaded into different inlets at the concentration of 7 million cells/mL and 10 million cells/mL, respectively. The viability dyes Sytox Green and Cell Event Caspase-3/7 Green were loaded along with the cells, and droplets were collected in the observation chamber. Droplets were generated at room temperature while collected into the observation chamber over a warm water bath at 37 °C. After collection, the droplets were incubated in a stage top incubator (Okolab) set at 5% CO_2_ and 37 °C temperature and observed using Nikon Ti2 microscope for 10 h. On average, the first timepoint of t = 0 h were imaged and analyzed after ± 10 min due to experimental procedures, i.e. the production, loading of droplets in the observation chamber and imaging each tile.

### Image acquisition and analysis

Fluorescence imaging was performed using a Nikon Eclipse Ti2 microscope, using a 10× objective and mCherry, DAPI, and FITC/YFP filters every hour. 15 × 15 tiles with resolution of 20,477 × 20,477 were framed per experiment. The images were viewed using NIS Element and Image J. Automated Image analysis was performed using custom-made in-build MATLAB script (Mathworks), DMALAB (available on request). The script generated droplet mask that was overlaid onto the fluorescence images, and each droplet was analyzed separately. Over 60,000 droplets per experiment were analyzed using this script. The cell division of the target or effector cell was not considered during the analysis. The output received are in terms of droplet index, cell count, fluorescence intensity and dead cell count. Detailed description of image analysis script is provided in “Result and discussion” section.

### Statistics and software

The Graphs were generated using GraphPad Prism 9.0.0. The results are expressed as mean ± SEM. Significant differences between two groups were analyzed by two-tailed unpaired Student’s t-test. P values < 0.05 were considered statistically significant.

## Results and discussion

### Droplet-based microfluidic platform to detect immune cell cytotoxicity

Cell-mediated cytotoxicity is a very dynamic and heterogeneous process since not all effector-target-cell interactions lead to targeted cell death^20^. Although molecular mechanisms mediating cytotoxicity have been extensively studied, the understanding of the determinants that govern the differential killing of specific target cells is yet elusive and often based on averaged and end-point readouts. Therefore, we developed a high-throughput cytotoxicity platform that combines (i) droplet generation and cell pairing using microfluidics, (ii) droplet immobilization for real-time microscopy, and (iii) automated image analysis to allow unbiased and high-throughput detection of cytotoxic events (Fig. 1A).

To automate cell tracking and the image analysis process, we labelled immune cells and target cells with different membrane permeable dyes. Next, we co-encapsulated them in water-in-oil droplets (~ 70 pL) using a 3-inlet microfluidic chip of 30 µm height, along with the viability dye and cell stimulus in the medium (Fig. 1B). An important aspect of our platform is the ability to track large numbers of droplets over time. For this purpose, we adapted a droplet immobilization chamber from Bounab et al. made by stacking two glass slides on top of each other that are glued together with thermo-responsive double-sided tape^23,24^. To ensure the sealing quality of the chamber, they were tested visually for deformation of the tape in the chamber and experimentally by testing the mobility of the droplets that were collected and were monitored over 10 h. We observed that the droplets barely moved over time and the chamber did not contain any air bubbles (Fig. 1C), indicative for an efficiently pressed and sealed chamber. The versatility of our approach lies in the flexibility of controlling the height of the chamber to allow immobilization of differently sized droplets.

In summary, our single-cell cytotoxicity platform allows for the imaging of over 50,000 droplets, thus allowing high-throughput monitoring of cellular interactions in a noise-free and controllable microenvironment in real-time.

### Highly tunable and controllable co-encapsulation of cells in droplets

Single cell cytotoxicity assays with primary cells, containing rare cell populations, emphasize the necessity to ensure efficient and controllable encapsulation. In practice, cell encapsulation does not always match the predicted values due to technical variables such as cellular sedimentation, and attachment or clumping of cells in the tubes. Over the years, there have been several design-related developments that have improved the encapsulation of single cells in droplets. Examples of such developments include the use of active encapsulation methods that utilize either acoustic or electrical forces for the generation of droplets containing single cells^25,26^. However, these methods have their own limitations in terms of bio-incompatibility and total expense compared to passive droplet generation techniques^27^.

**Figure 2:**
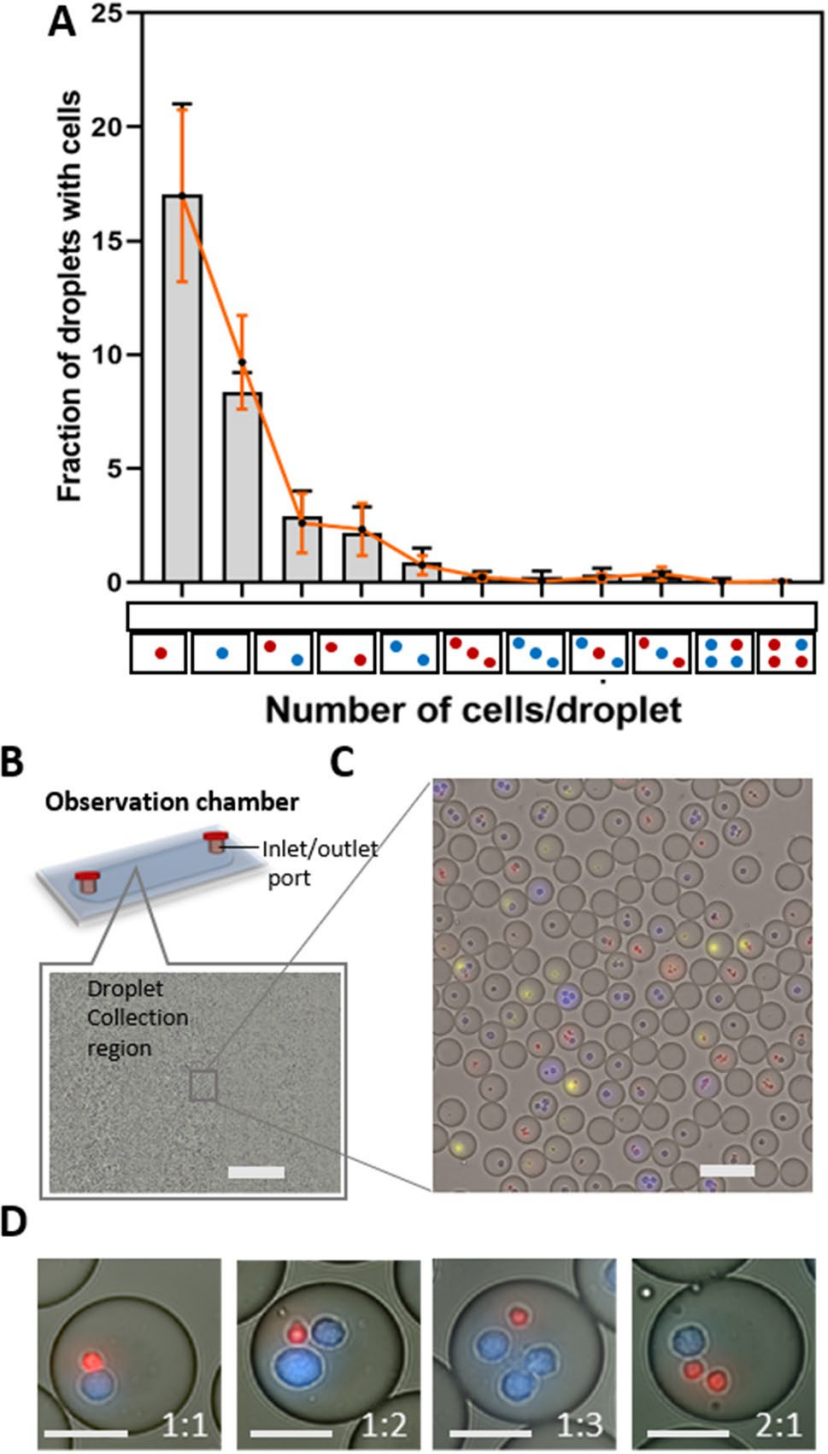
Cell encapsulation and pairing in droplets. (**A**) Graph showing the predicted fraction of droplets containing a different combination of cells illustrated on the x-axis according to Poisson distribution (red solid line) and the observed fraction of droplets containing given combinations of cells (bar diagram) at 7 million/mL NK cells (red) and 10 million/mL K562 cells (blue); n = 3 independent experiments; red and black dots represents mean value for given combination for Poisson distribution and observed distribution respectively. Error bars represents standard error of mean. (**B**) Schematic and Macroscopic view of the observation chamber. The image represents around 229 mm^2^ viewing area of the chamber, allowing to image ~ 100,000 droplets of 70 pL volume; scale bar = 1 mm. Droplets without cells are not shown in the figure. (**C**) Magnified segment in the observation chamber showing distribution of different cells in droplets; scale bar = 100 µm. (**D**) Visualization of different E:T ratios in droplets at the cell seeding concentration of 7 million/mL and 10 million/mL for effector and target cells, respectively; scale bar = 25 µm.

To combat those limitations, we utilized the tip-loading method that we had optimized before, ensuring efficient cellular encapsulation that closely matched the Poisson distribution^22^ (Fig. 2A and Supplementary Fig. 2A–D). The rate of cell arrival at the junction is a function of cell seeding density. Therefore, by varying the concentrations of cells, we can maximize cell pairing. By using different cell concentrations for effector and target cells, different Effector:Target (E:T) ratios in droplets were obtained. We aimed for the highest 1:1 (E:T) encapsulation ratios while avoiding excessive cell clumping, which we achieved using concentrations of 7 and 10 million cells/mL for effector and target cells, respectively. At this concentration, we showed that around 3% (~ 2000 droplets) of the total fraction of droplets had 1:1 cell pairing (Fig. 2A). At concentrations above 10 million/mL, the encapsulation became unstable because of the large number of cells clustering at the inlet and resulting in channel blockage, (Supplementary Fig. 3A,B).

Until now, cell pairing efficiency in droplets at high throughput had been the limiting factor in developing a platform that combines real-time microscopy with cellular interactions-based assays. Several groups have developed a variety of approaches such as hydrodynamic trap-based or microwell/nanowell-based platforms to tackle this problem^3,28,29^. Even though these platforms achieved high pairing efficiencies and dynamic monitoring, they failed to exclude paracrine signalling between neighbouring cells and lack high throughput, important factors that need to be considered when studying and understanding functional heterogeneity in immune cells^30^. Furthermore, the droplet-based cytotoxicity platform developed by Sarkar et al. utilized a docking array-based system, allowing the visualization of around 4000 droplets and the platform developed by Antona et al. utilized spherical traps, allowing the visualization of around 6500 droplets per experiment^20,21^. These studies analyzed approximately 100 droplets to study the cellular interaction in between NK cells and target wells. Since immune cells can be highly heterogeneous in their effector functions, throughput is vital to discover small and distinct subsets. So far, to the best of our knowledge, no other droplet-based cytotoxicity platform has been able to meet that requirement in such a way as our platform, allowing the visualization of around 60,000 droplets per experiment (Fig. 2B,C). Among those droplets, this platform allows to analyse 20,000 droplets with cells including cell pairs at different E:T ratio thereby allowing to do proper statistical analysis and even identification of rare cells (Fig. 2D). Droplets with only either effector or target cells serve as an internal negative control for viability (Supplementary Fig. 4A,B). Droplets with multiple effector cells encapsulated with one or more target cells are used to examine the effect of intercellular interactions and paracrine stimulation. Finally, the droplets with only one effector cell and multiple target cells are monitored to examine the so-called serial killing effect of effector cells. Concluding, our droplet-based microfluidic platform allows for easy, highly controllable effector-target co-encapsulation efficiencies, including proper biological controls and the possibilities to examine the effects of paracrine signaling and serial killing in parallel.

### Live fluorescent cell labelling allows for cell tracking and identification of cytotoxic events

**Figure 3:**
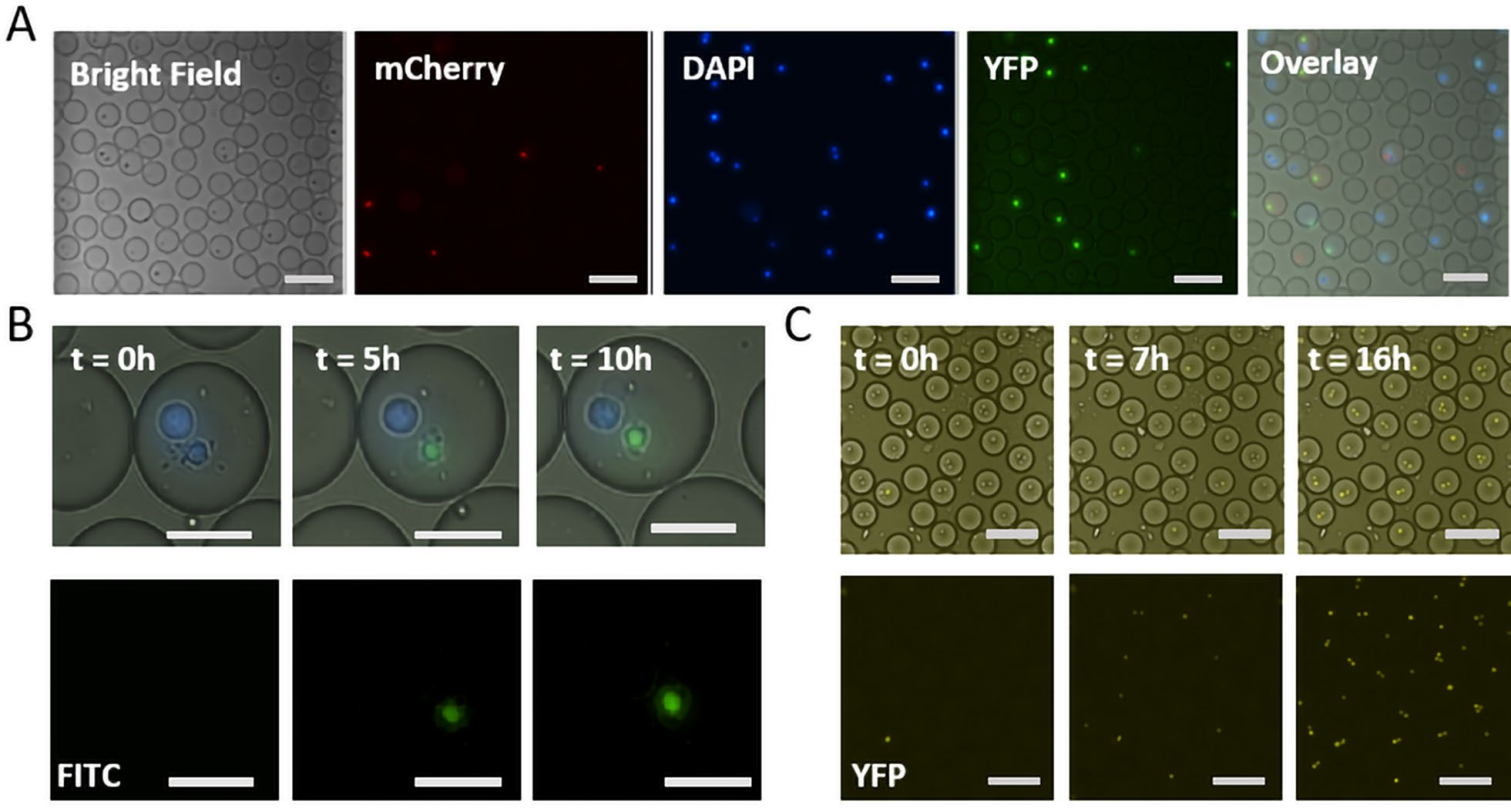
Real-time viability tracking. NK cells and target cells (here K562 cells and Jurkat T cells) were labelled with Calcein Red AM (5 µM) and Cell Tracker Blue (10 µM), respectively. The viability dyes Sytox Green (2.5 µM) and Cell Event Caspase 3/7 (7 µM) were encapsulated in droplets with cells and culture medium. (**A**) Image showing different cells in droplets that are monitored using mCherry for NK cells, DAPI for K562 cells, and YFP/FITC for dead cells; scale bar = 100 μm. (**B**) K562 cells were labelled with Cell Tracker Blue (25 µM) and co-encapsulated with Sytox Green as viability marker. The signal remains stable over 10 h; scale bars = 25 μm (**C**) Jurkat T cells were encapsulated in droplets along with (Cell Event Caspase3/7 (7 μM) and 5% DMSO to induce apoptosis. In presence of DMSO, the cell undergoes apoptosis after 6 h, and the staining remains stable over 10 h; scale bars = 100 μm.

A crucial aspect of the droplet-based cytotoxicity platform is the accurate monitoring of cell viability in droplets. Additionally, it is also important to trace if either an effector cell or a target cell died once a cytotoxic event is measured (Fig. 3A). To trace cells over time, they were labelled with the commonly used live cell imaging dyes, Cell Tracker Blue, and Calcein Red AM. The latter is also used as a viability marker in some cytotoxicity-based studies, however, the possibility of signal loss caused by membrane pumps can make it difficult to use this dye for real-time assays with longer incubation times^31,32^. The stability and toxicity of the dyes were tested using our custom-made DMLAB script over time. We observed that the signal from Cell Tracker Blue labelled cells remained stable, whereas the signal from Calcein Red AM reduced gradually over time without inducing toxic effects (Supplementary Fig. 5A–D).

To achieve an accurate and dynamic readout of the viability of cells in droplets, two viability markers were tested: Sytox Green, for necrotic and late apoptotic events, and Cell Event Caspase, to detect early apoptotic event. In droplets, we observed, for both candidate dyes, that they can dynamically stain apoptotic cells, while remaining stable over time, therefore being the perfect candidate to also detect early and late apoptotic events (Fig. 3B,C and Supplementary Fig. 6A,B).

These results show that different dyes can be used together with our script to trace and identify cytotoxic events in real time.

### Script for robust, automated, high throughput image analysis of cytotoxic events

The droplet-based cytotoxicity assay yields fluorescence images with thousands of droplets at multiple time points. To maintain the high-throughput character of the assay, and to gain accurate results, an automated readout of these images is pivotal. For a complete automation of the image analysis, certain requirements were fulfilled. First, the script should be able to track the droplets over several hours. Second, the script should be able to distinguish the different cell types and corresponding numbers of cells in each of the tracked droplets. Third, it should be able to accurately track the change in viability signal for each cell type to connect the viability signal to the corresponding cell, which could either be the effector or target cell. To this end we developed a custom-made In-Droplet Viability Analysis script, or DMALAB script, to allow for automated detection of thousands of cells, corresponding cytotoxic events, and full control over the image processing procedure.

**Figure 4:**
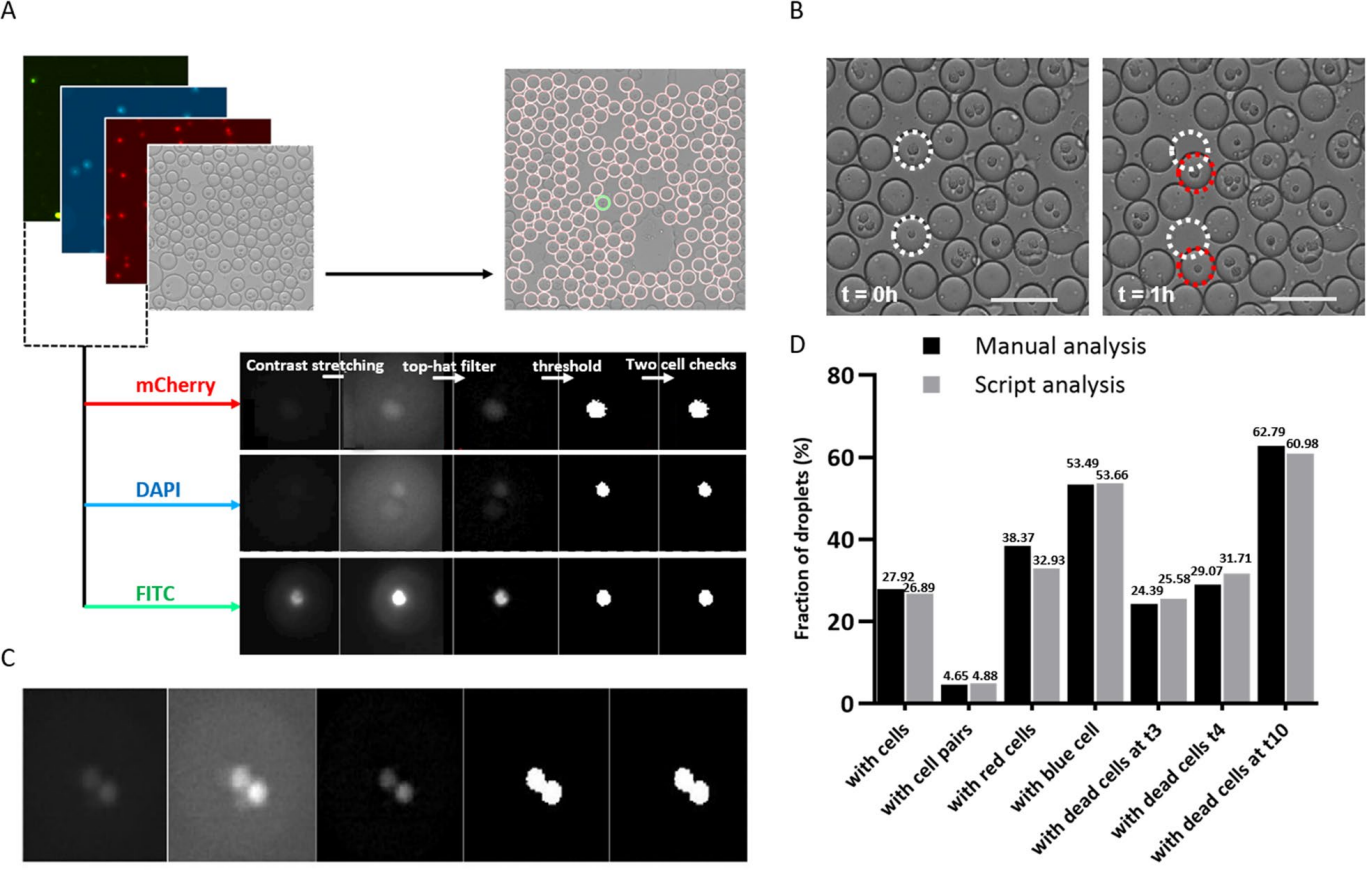
Image processing and analyzing steps in MATLAB script. (**A**) Image processing by stacking different channels, droplet tracing, and detection of cells over each channel. (**B**) Droplet movement over one hour. An adjustable amount of movement is allowed by the script, but if this allowed movement is more than the droplet radius, the risk exists of detecting a different droplet at the next time point. Droplet detection over time is done by comparing the coordinates of the center of the droplet between two consecutive time points and selecting the closest center within the allowed movement range. (**C**) Evaluation of the script for cells recognition over different channels, here two cells labelled with cell tracker blue are identified in DAPI channel). (**D**) Validation of the script by comparing differences in cell distribution, cell pairing, and dead cell identification at three different time points (t = 3 h, t = 4 h, and t = 10 h) in-between script generated data with manually counted data.

In short, the image analysis script consists of three steps: (i) droplet tracing (BF), (ii) fluorescence image pre-processing (fluo), (iii) and cell counting (ncel; Fig. 4A). The process starts with detecting individual droplets in the bright field channel using the commercially available “imfindcircle” function. The script allows to optimize the settings for the droplet identification, which are based on the shape, size, and immobilization of the droplets. Using contrast stretching, the edges of the individual droplets get identified, allowing the accurate monitoring of moving droplets over time. Next, each droplet will be given an index number, while the coordinates determine the droplet position. The script allows for the control over contrast stretching parameters and tracks and removes the moving droplets, thus ensuring the accuracy of droplets detection over different time frames (Fig. 4B). After the script has traced the individual droplets, the next step is to detect the individual cells per droplet. To facilitate the cell detection in the droplets, we prelabeled the cells with fluorescent dyes. With the objective of obtaining a binary image that represents the object (here cells), the fluorescent images are preprocessed by cropping, applying a top hat filter, setting the intensity of non-droplet pixel to zero and applying the threshold to get the binary image (Fig. 4C). The final part of the analysis consists of cell counting within droplets and the detection of changes in fluorescence. Finally, all objects can be counted, and the change in fluorescence can be detected at sequential time points.

To validate the performance of the script, Overnight activated NK cells in presence of 50 ng/ml IL2 were encapsulated with K562 cells in the droplets and the results from manual and automated analysis in terms of droplets count, cell distribution, cell pairing, and viability of cells at random time frames were compared. Upon comparing, we obtained coherent results between the two methods with a maximum deviation of only 2% (Fig. 4D and Supplementary Fig. 7A,B). In case of doubts, there is also an inbuilt droplet trace function that allows for the visualization of the individual droplets, for all fluorescent channels, and the possibility to exclude droplets from downstream analysis if needed. Altogether, our script enables high-throughput analysis of images with large amounts of data by automatically detecting cells and their viability in droplets. It gives a high level of control over the analysis parameters, making it easily applicable in various kinds of functional droplet-based assays.

### Dynamic nature of NK cell-mediated killing of tumor cells

**Figure 5:**
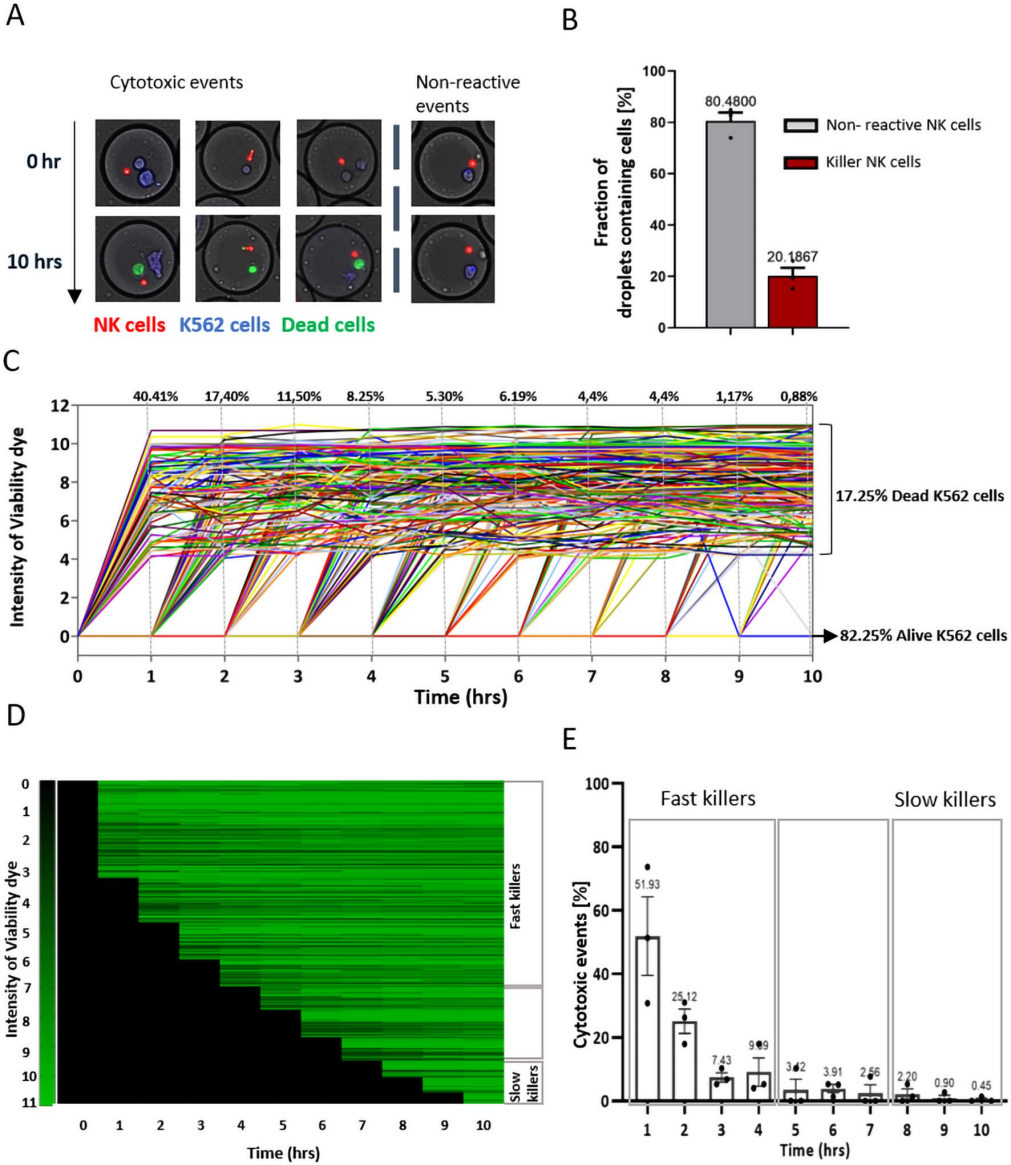
NK cytotoxicity in real-time. (**A**) Microscopic overview of NK cell-mediated cytotoxicity in droplets. NK cells were labelled with Calcein Red AM dye (red cells) and K562 cells with Cell Tracker Blue (blue cells) and paired together in 70 pL droplets in presence of viability dye (Sytox green and Cell event Caspase 3/7). They were then incubated at 37 °C and 5% CO_2_ for 10 h. Image acquisition was done at an hour interval. Over the interval of 10 h, NK cells are interacting with target cells, either, with (left) or without inducing cytotoxicity (right). The cells that die are stained with the viability dye and thus turn green. The live cell retains the original color. (**B**) Bar diagram representing killer fraction of NK cells in droplets; data analysis performed on E:T 1:1; n = 3 independent experiments; error bar represents standard error of mean. (**C**) A representative experiment showing intensity of the viability for K526 cells upon interaction with NK cells plotted over time. Each line represents individual cells; n = 66,600 total droplets with 20,860 droplets containing cells. The graph does not include the droplets with 0, or 1 cell and droplets with dead cells at t = 0 h. An alive cell gives the intensity of viability dye as “0”, cells with values above zero are considered as dead. (**D**) Heat Map showing the dynamics of cytotoxicity within an experiment. Each line represents individual cells. The viability was assessed over the period of 10 h. (**E**) Graph representing the dynamics of cytotoxic events in droplets for different donors. The dynamics were determined for the killer fraction of NK cells, thus showing the percentage of the fast and slow killer population in NK cells; data analysis performed on E:T 1:1; n = 3 independent experiments; error bar represents standard error of mean.

NK cells are well known for the efficient identification of their targets to induce killing without prior antigen sensitization, making them an ideal target for anti-cancer immunotherapies^33^. They identify target cells that lack or have reduced expression of MHC-1 molecules and subsequently kill upon balanced signaling between activation and inhibitory receptors^34^. The use of several receptor interactions and signaling pathways makes NK cells a very complex and heterogeneous cell type^35^. The cytotoxic interactions are both effector-cell as well as target-cell dependent, which further enhances the variation in responses mediated by both types. Therefore, to address functional heterogeneity in the NK cell compartment, we co-cultured them with K562 as target cells. K562 is a myelogenous leukemia cell line that lacks MHC-1 expression and therefore is an attractive target to further characterize NK cell-mediated cytotoxicity. The effector (NK cells) and the target (K562) cells were paired together in 70 pL droplets, allowing them to interact. The study by Antona et al. showed that cells in a confined environment, such as droplets, increase the chances of cellular interaction^21^. However, we observed that only around 20% of NK cells were potent killers, while the large majority were not able to induce killing at all during the 10 h of incubation, which is in agreement with the two earlier micro-well based studies by Vanherberghen et al.^2^ and Gudeval et al^3^ (Fig. 5A,B). In contrast to the work described by Sarkar et.al., we did not observe 100% NK cell-mediated killing in droplets, which we believe is explained by the characteristics of the utilized dye, which includes the sensitivity of being actively pumped out by the target cell^20^. Interestingly, our results reveal heterogeneity within the NK cell compartment with only part of the population displaying cytotoxic behavior. This indicates that only a small percentage of NK cells kill, whilst the majority are either inactive, very late killers (beyond our time frame of 10 h) or require passive support from paracrine signaling from the other cells to be able to induce cytotoxic ability.

Additionally, the dynamics of NK cell mediated cytotoxicity of K562 cells was monitored using our platform. During the incubation, we observed that different NK cells demonstrate different potential to kill. In a representative experiment, we analyzed 66,600 droplets in total (18,320 droplets with cells), of which 2052 droplets contained the desired cell pairing (Fig. 5C,D). By plotting the intensity of viability dye over incubation time, we showed the dynamics of cellular interaction between NK cells and K562 cells. Our findings from this experiment revealed that 17.25% K562 cells were dead whereas 82.5% K562 cells were alive despite being incubated together with NK cells. We also observed that a large fraction of cytotoxic NK cells (77%) induce cytotoxicity within the first four hours of interaction. 40% of the NK cells provided lytic hits within first hour of interaction while the incident of cell death decreased over time and was minimal after 10 h (0.88%). These findings were consistent with other donors as well. Of all the NK cells that have the potential to kill K562 cells, approximately 86% delivered a lytic hit within four hours of incubation, and we refer to them as fast killers. Among these fast killer cells, 52% of cells initiated killing within 1 h of incubation (Fig. 5E). 25% of NK cells were able to mount the cytotoxic ability in the second hour of incubation and the number went down subsequently over time. In contrast, K562 in absence of NK cells had higher viability (around 95%) over 10 h and most of the target cell death occurred only at the later phase of the incubation (Supplementary Fig. 4B). These results suggested that a majority of NK cell population showed spontaneity on killing their target cells upon recognition.

The data presented here supports the classification of NK cells based on their cytotoxic ability and the dynamics at which they kill their target cells^2^. Even though the time of target cell lysis varied considerably over different time point during the course of incubation, our result supports the notion of the existence of a NK cell subpopulation that induces killing as early as 30 min after interaction with a target cell^2,36,37^. The fast and slow killing ability of NK cells could be coupled to the granzyme dependent and independent mechanisms by which NK cells induces cell death^38^. Furthermore, The size and content of the lytic granules along with the secretion and quantity of degranulation events can also be associated with the kinetics of target cell killing by NK cells^39^. We can relate these variations in killing capacities of NK cells to functional heterogeneity within NK cell population. However, the potential cause underlying these variations was not explored in this research and requires further investigation.

Some NK cells have the capacity to kill multiple target cells, referred to as serial killers^2,40^. For years, translational researchers that want to use NK cells for immunotherapy have aimed to harness this extremely relevant and promising feature^2,41^. Although this search goes beyond the scope of this study, we investigated the pairing distribution and the cytotoxicity induced by different E:T ratios (Supplementary Fig. 8A) to characterize serial killing ability of NK cells. Even with a limited sample size, we could identify several instances (around 2.5% of droplets with the combination of multiple K562 and a single NK cells; data not shown) where a single NK cell could kill two target cells, supporting the previously observed serial killing ability of NK cells^41^. Future studies will be needed to assess the serial killing potential of NK cells, which can be achieved by minimal adaptations to our experimental workflow.

Concluding, our droplet-based microfluidic platform allows for easy, highly controllable effector-target co-encapsulation efficiencies, including proper biological controls and the possibilities to examine the effects of paracrine signaling and serial killing in parallel.

## Conclusion and outlook

In this paper, we have presented an integrated platform that allows for monitoring and decoding cellular interactions between immune and tumor cells in high throughput. This methodology was built on the already available and broadly utilized principles of cytotoxicity assessment and live-cell imaging. Here we combined (i) the assessment of different E:T ratios, (ii) a dynamic readout, and (iii) an automated image analysis script that allows an unbiased and high-throughput detection of cytotoxic events. Besides, this cytotoxicity platform is designed to be easily adjusted according to different experimental needs, allowing the assessment of cytotoxicity in a wide variety of both immune cells and target cells.

We show that NK cell cytotoxic effector functions are highly dynamic and heterogeneous, which could otherwise not have been studied with conventional assays. The value of this microfluidic setup is its simplicity, both in fabrication and experimental workflow. Future minor modifications to adapt this platform for droplet sorting and barcoding will also allow the use of this setup for omics-based studies, thereby opening new avenues of research in cellular and functional diversity. Apart from NK cells, there are several cell types including CTLs, macrophages, and killer dendritic cells that can exhibit cytotoxic abilities, and have been widely explored for immunotherapeutic purposes. In vitro cytotoxicity assays have been an important assessment tool for measuring the maturation and functional activities of these immune cells. Therefore, a comprehensive evaluation of cell-mediated cytotoxicity can become an important parameter to correlate the treatment with the clinical outcome. Since we deliberately designed our platform to allow flexibility, other cell types can easily be studied without the need for modifications. Therefore, this platform can be easily adapted to the abovementioned cell types and their killing dynamics can be assessed for clinical as well as for research purposes.

## Supplementary Information

Supplementary Figures.
