# Supplementary material for: An automated real-time microfluidic platform to probe single NK cell heterogeneity and cytotoxicity on-chip
Source: Sci Rep. 2021 Aug 24;11:17084. doi: 10.1038/s41598-021-96609-9 (PMC8385055; doi:10.1038/s41598-021-96609-9)
Supplement: Supplementary file 1 — Supplementary Figures. [file 41598_2021_96609_MOESM1_ESM.pdf]

An automated real-time microfluidic platform to probe single NK cell heterogeneity and cytotoxicity on-chip

Nikita Subedi[1,2], Laura C. Van Eyndhoven[1,2], Ayla M. Hokke[1,2], Lars Houben [1,2], Mark C. Van Turnhout[3], Carlijn V.C. Bouten[2,3], Klaus Eyer[4], Jurjen Tel[1,2] *

[1] Laboratory of Immunoengineering, Department Biomedical Engineering, Eindhoven University of Technology, Eindhoven, Netherlands
[2] Institute for Complex Molecular Systems (ICMS), Eindhoven University of Technology, Eindhoven, Netherlands
[3] Soft Tissue Biomechanics and Tissue Engineering, Department Biomedical Engineering, Eindhoven University of Technology, Eindhoven, Netherlands
[4] Department of Chemistry and Applied Biosciences, ETH Zurich, Zurich, Switzerland

* corresponding author j.tel@tue.nl

Supplementary figures

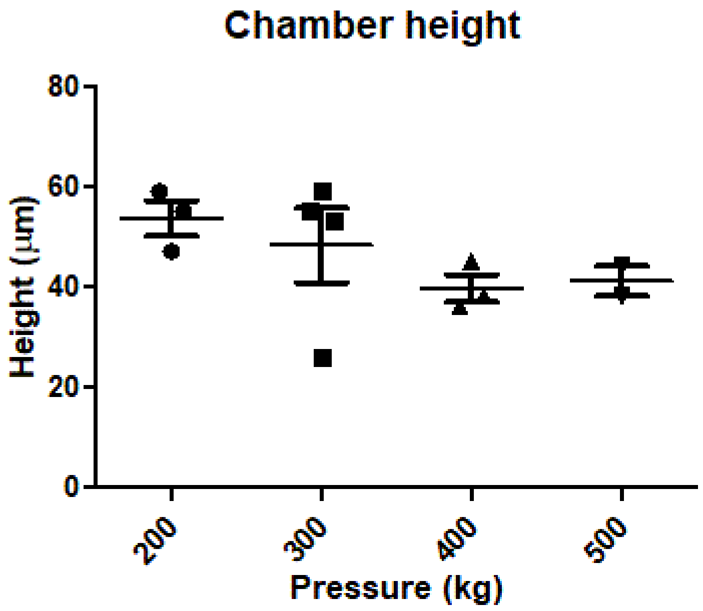

Supplementary Figure 1. Graph showing effect of different pressure loads (200 kgs, 300 kgs, 400 kgs, and 500 kgs) on the height of the collection chamber; Error bar represents standard error of mean.

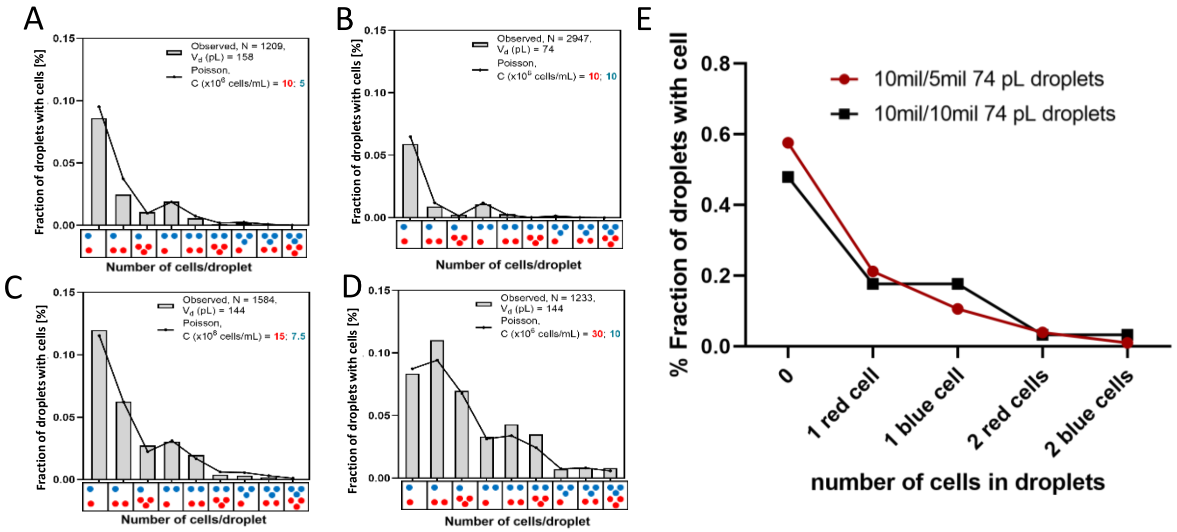

Supplementary Figure 2. Co-encapsulation efficiency of effector cells and target cells at various seeding densities. Graphs showing the predicted fraction of droplets containing the combination of cells illustrated on the x-axis according to Poisson distribution (lines) and the observed fraction of droplets containing those combinations of cells (bars) at various seeding densities. The seeding densities were: (A) $10*10^6$ red cells/mL and $5*10^6$ blue cells/mL, (B) $10*10^6$ cells/mL for both cell types, (C) $15*10^6$ red cells/mL and $7.5*10^6$ blue cells/mL, (D) $30*10^6$ red cells/mL and $10*10^6$ blue cells/mL. (E) line graph representing the fraction of droplets with and without cells (irrespective of cell pairing) at different concentration.

A B

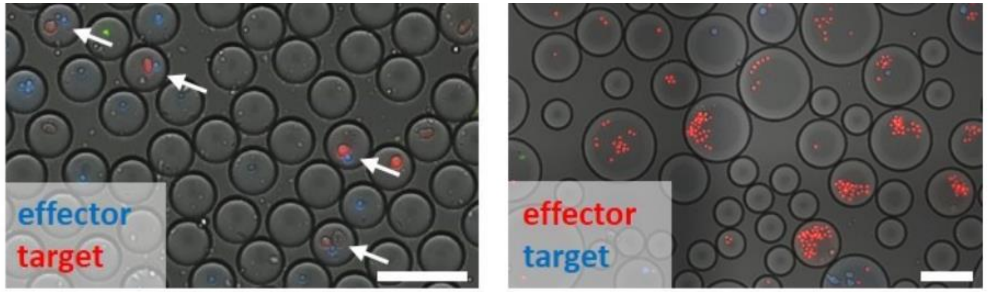

Supplementary Figure 3. Overview of droplets containing different numbers of cells. The seeding densities in this experiment were 10*10^6 cells/mL for both cell types. Droplet with cell pairing are indicated with arrows. (B) Cell clumps in polydisperse droplets. Here, the seeding density of the effector cells was 15*10^6 cells/mL, and the seeding density of the target cells was 7,5*10^6 cells/mL. Scale bars are 100 µm.

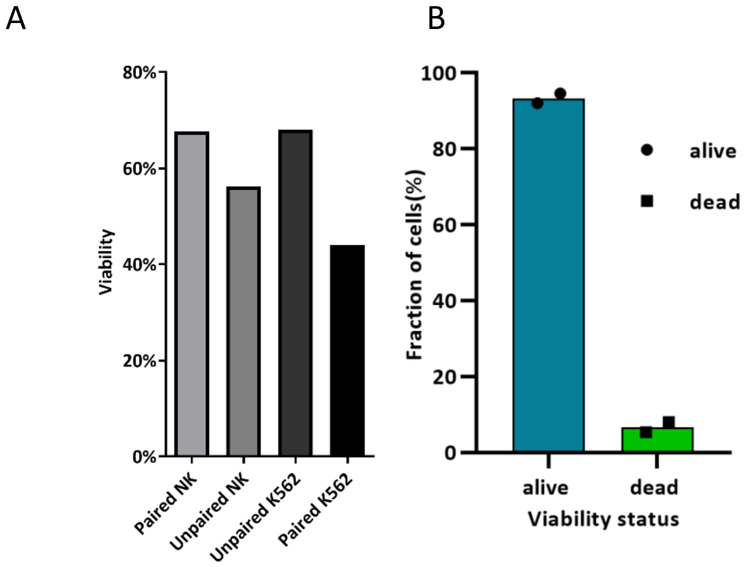

Supplementary Figure 4. Distribution of viabilities within droplets. (A): The total counted viabilities of NK cells and target cells (n=1000 droplets). The graph depicts the experiment with NK cells paired together with K562 were all E:T ratios were taken together to show the difference between unpaired and paired cells. (B): the viability of K562 cells in droplets; The graph depicts the experiment with only K562 in the droplets. Error bar represents Standard error of mean; n=2,; t=10h.

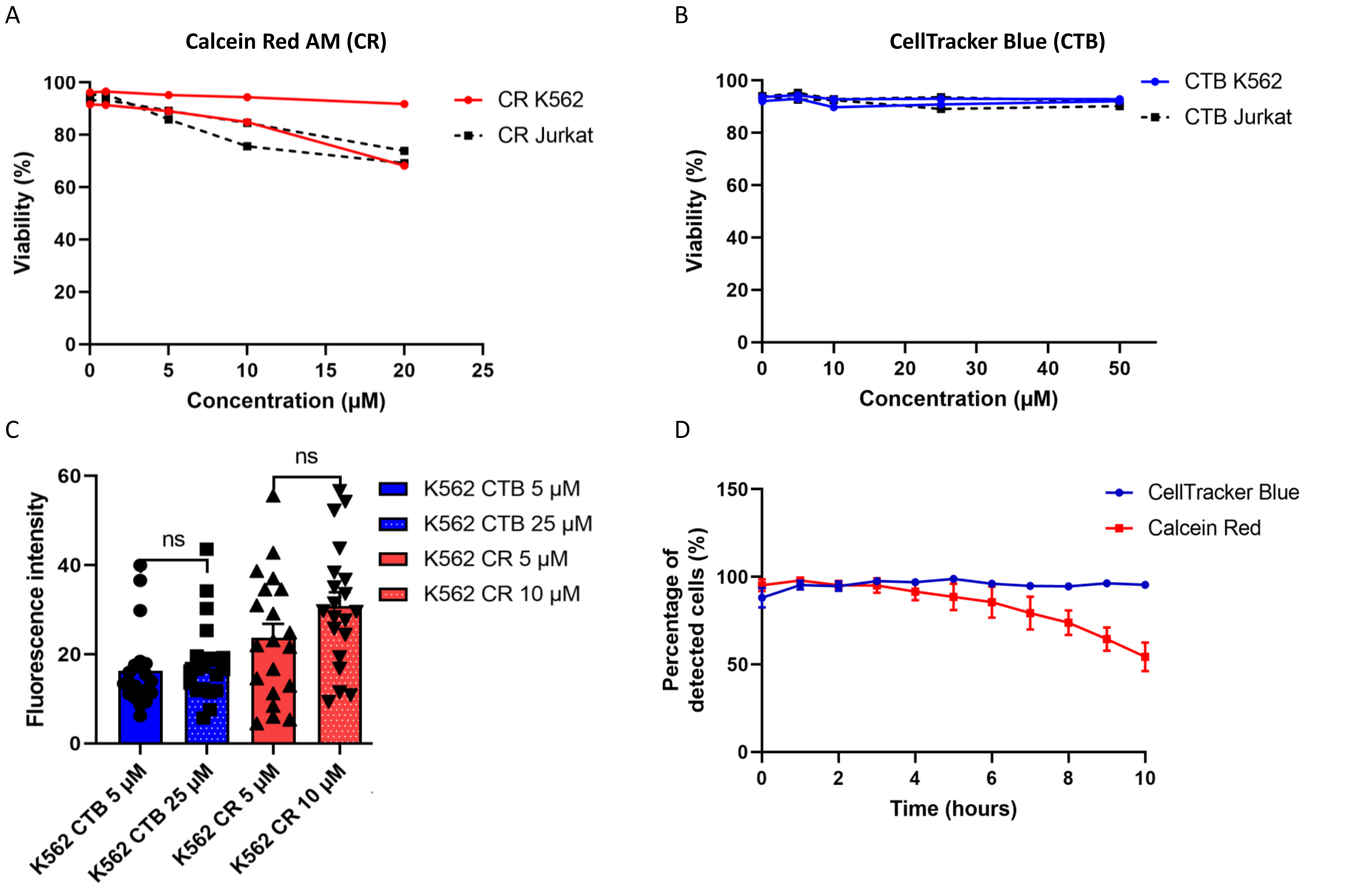

Supplementary Figure 5. Characterization of the use of Calcein Red AM (CR) and CellTracker Blue (CTB). (A-B) K562 cells and Jurkat cells were labelled with different concentrations of CellTracker Blue and Calcein Red AM, washed, and cultured overnight. Necrosis and apoptosis was assessed using flow cytometry. n=2 for both graphs. (C) K562 cells were labelled with different concentrations of CellTracker Blue (both cell types) and Calcein Red AM (K562 cells), washed, and encapsulated in droplets. Fluorescent signal of 20 randomly sampled cells was then measured using ImageJ and corrected for background. Significance was tested with unpaired Student's t-test, two-tailed. (D) Jurkat cells were labelled with Calcein Red at 5 μM and CellTracker Blue at 5 μM and were encapsulated in droplets. The DMLAB script was then used to detect cells. The percentage of cells that was detected over time over the maximum number of cells that was detected is depicted in the graph. n=3 for both cell dyes; Error bar represents SEM.

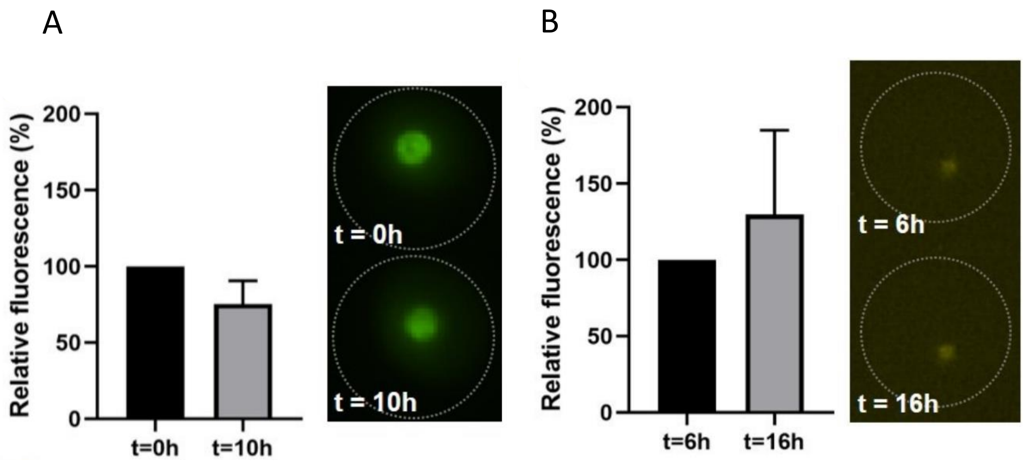

Supplementary Figure 6. Stability of viability dye over time (A) Sytoxgreen (Nucleic acid binding) dye stability was measured on K562 cells that were labelled with CR at a concentration of 10 µM; n=20; Error bar represents SD. (B) Caspase detection reagent signal intensity was measured on Jurkat cells (not labelled with CR); n=20; Error bar represent SD.

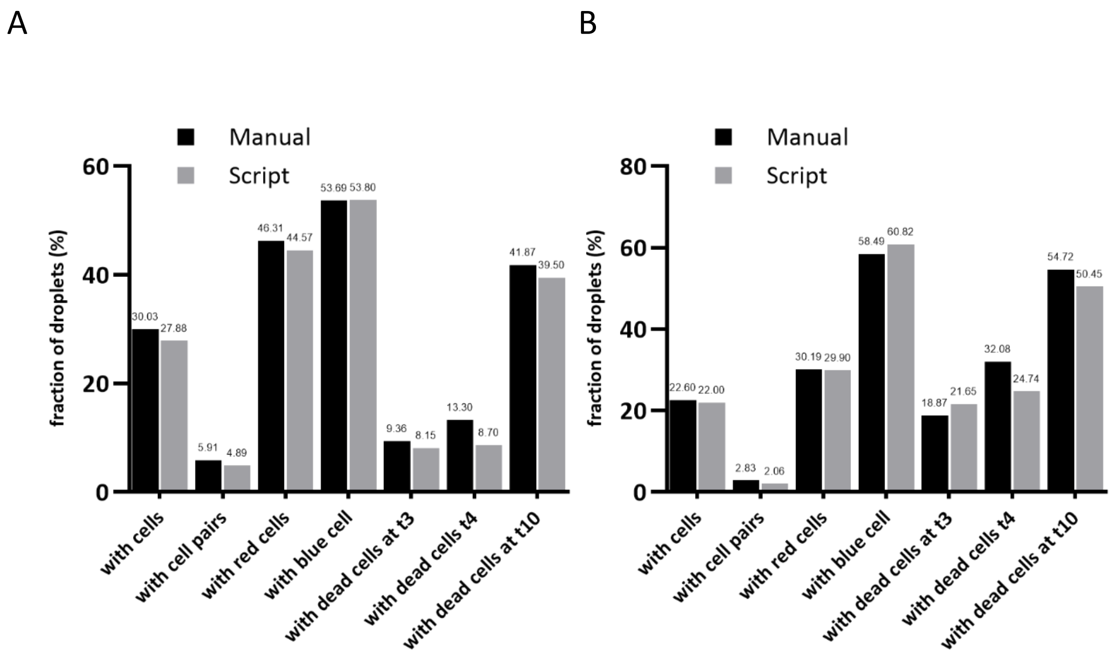

Supplementary Figure 7. Distribution of viabilities within droplets. (A and B): Validation of the script by comparing differences in cell distribution, cell pairing, and dead cell identification at 3 different time points (t=3, t=4, and t=10) in-between script generated data with manually counted data.

A

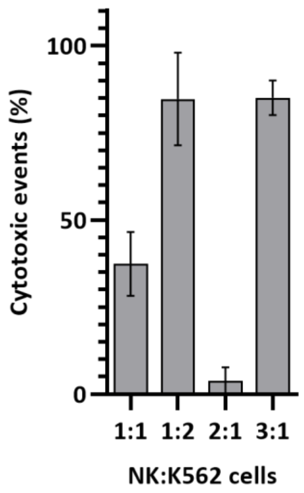

Supplementary Figure 8. Distribution of different E:T ratio in droplets: Difference in cytotoxicity at different cellular distributions (E:T 1:1,1:2,2:1,3:1);n=3 (for representative n=1 total droplets pair analyzed 1143; for 1:1 1066 droplets; 1:2 13 droplets ; 2:1 49 droplets; 3:1 15;); error bar represents standard error of mean.
